# The Association Between Alopecia Areata and Iron Deficiency Anemia: A Large-Scale Population-Based Case–Control Study

**DOI:** 10.3390/jpm16060283

**Published:** 2026-05-26

**Authors:** Batya Davidovici, Yarden Drutin, Amir Ben-Tov, Daniel Mimouni, Yonit Wohl

**Affiliations:** 1 Faculty of Medicine, Tel Aviv University, Tel Aviv 6997801, Israel; 2 Division of Dermatology, Rabin Medical Center—Beilinson Hospital, Petach Tikva 4941492, Israel; 3 Pediatrics Department, Dana-Dwek Children’s Hospital, Tel Aviv Sourasky Medical Center, Tel Aviv 6423906, Israel; 4 Maccabi Health Services, Tel-Aviv 6812509, Israel; 5 Kahn Sagol Maccabi Research and Innovation Center, Maccabi Healthcare Services, Tel-Aviv 6423906, Israel; 6 Faculty of Medicine, Ariel University, Ariel 4070000, Israel

**Keywords:** alopecia areata, iron deficiency anemia, autoimmune disease, comorbidity, case–control study

## Abstract

**Background**: Alopecia areata (AA) is a chronic autoimmune disorder characterized by non-scarring hair loss and commonly coexists with other autoimmune diseases, suggesting systemic immune dysregulation. An association between AA and iron deficiency anemia (IDA) has been proposed, though prior studies have yielded conflicting results. **Objective**: Our aim was to examine the association between AA and IDA in a large, population-based cohort. **Methods**: We conducted a retrospective case–control study using data from a health maintenance organization (HMO) serving approximately 2.35 million individuals. We included 33,401 AA patients diagnosed between 2005 and 2019, matched 1:2 by age and sex with 66,802 controls without AA. Diagnoses of AA and IDA were confirmed through clinical coding by board-certified dermatologists and primary care physicians. Statistical analyses employed Kruskal–Wallis, Pearson’s χ^2^, and Fisher’s exact tests. **Results**: IDA prevalence was significantly higher in AA patients than in controls (15% vs. 10%; OR 1.61, 95% CI: 1.55–1.67; *p* < 0.01). IDA preceded AA in 40% of cases, followed AA in 58%, and occurred concurrently in 2%. Limitations: The retrospective design limits causal inference. **Conclusion**: This study reinforces an association between AA and IDA, highlighting the need to assess and manage IDA as a potential treatable comorbidity in AA patients.

## 1. Introduction

Alopecia areata (AA) is a common chronic autoimmune disease. It is the second most common cause of hair loss following androgenetic alopecia, affecting up to 2% of the global population [1]. AA presents with non-scarring hair loss, commonly in sharply demarcated patches on the scalp, face, or body. The disease may progress to more severe forms, including alopecia totalis and universalis, which can have a profound psychological and social impact [1,2,3]. Genetic susceptibility, environmental triggers, and immune system dysregulation collectively contribute to the onset and progression of AA [4,5].

The pathological hallmark of AA is an immune-mediated attack on the immune privilege location of the hair follicles during the anagen phase, predominantly driven by T lymphocytes infiltrating the follicular environment. Although the immunological mechanisms are complex, it is widely accepted that inflammatory T-cell responses and impaired immune regulation underlie the disease process [6].

AA is increasingly recognized as part of a broader systemic autoimmune profile. Notably, autoimmune thyroid diseases such as Hashimoto’s thyroiditis and Graves’ disease are frequently observed comorbidities, with thyroid dysfunction present in up to 30% of AA patients [7,8]. Additional autoimmune conditions, including vitiligo, systemic lupus erythematosus, rheumatoid arthritis, and celiac disease, are also reported at higher prevalence among AA patients [8,9]. Individuals with AA also show increased rates of atopic diseases such as asthma, allergic rhinitis, and eczema, pointing to a general predisposition toward immune dysregulation [8,10].

Recent research has increasingly focused on the potential relationship between AA and iron deficiency anemia (IDA). While earlier studies yielded conflicting conclusions [11,12], more recent large-scale investigations suggest that IDA may be significantly associated with AA [13]. Iron plays a critical role in immune regulation, and its deficiency may exacerbate immune dysregulation and sustain inflammation [14]. Chronic iron deficiency may contribute not only to disease severity but also to disease initiation in susceptible individuals.

In this study, we sought to investigate the association between AA and IDA in a large, population-based cohort. Our aim was to clarify this relationship and contribute to the evolving understanding of AA as a systemic autoimmune disease. Clarifying this association may also have implications for personalized medicine approaches in dermatology, as identification of modifiable systemic factors such as iron deficiency could help refine individualized diagnostic evaluation and management strategies for patients with AA.

## 2. Methods

We conducted a retrospective population-based case–control study using data from Maccabi Healthcare Services (MHS), the second-largest state-mandated health fund in Israel, covering approximately 2.35 million individuals (26% of the national population).

All patients diagnosed with AA between January 2005 and December 2019 were identified using established diagnostic codes confirmed by board-certified dermatologists. Each AA case was matched to two healthy controls by age and gender. Patients with iron deficiency anemia were identified based on the presence of specific ICD-9 diagnostic codes recorded in their medical records. The following ICD-9 codes were used to define cases of iron deficiency anemia by primary care physicians: 280.0, 280.1, 280.8, 280.9. These codes were extracted from electronic health records and served as the primary criteria for case identification. Although it provided support for the diagnosis, laboratory confirmation was not mandatory.

The study was conducted in accordance with the Declaration of Helsinki, and approved by the Maccabi Healthcare Services Institutional Review Board (protocol code 0131-20 and date of approval 8 January 2020).

### Statistical Analysis

Statistical analyses were performed using SPSS version 28.0 (IBM, Armonk, NY, USA). The Kruskal–Wallis test was applied to continuous variables, while Pearson’s χ^2^ test or Fisher’s exact test were used for categorical variables. A logistic regression model was utilized to estimate odds ratios (ORs) and 95% confidence intervals (CIs), adjusting for potential confounders. A *p*-value of <0.05 was considered statistically significant.

## 3. Results

The study cohort included 33,401 patients with AA and 66,802 matched controls. The mean age of AA onset was 29.9 years, with 56.5% male and 43.5% female patients. The vast majority (>80%) were Israeli-born (Table 1).

Out of the total cohort, 11,789 patients (11%) had IDA.

Compared to the control patients, IDA was diagnosed in 15% of AA patients (5080 individuals) versus 10% of controls (6709 individuals), demonstrating an increase of 60% in AA patients, a statistically significant association (odds ratio = 1.61, 95% CI: 1.55–1.67, *p* < 0.01).

In a subgroup analysis by age group (Table 2), differences remained significant in all age groups, especially in AA patients aged 18–40 who exhibited the most prominent increase in IDA prevalence (odds ratio = 1.65, 95% CI: 1.56–1.76, *p* < 0.01).

The consistent association observed across all age groups, with the strongest effect among young adults, suggests that age-specific risk profiling may help to identify AA patients who could particularly benefit from targeted screening for iron deficiency as part of a more individualized clinical evaluation.

Temporal analysis showed that in 40% of cases, IDA preceded AA diagnosis. In 58% of cases, AA was diagnosed before IDA, and in 2% of cases, both were diagnosed within a three-month window.

## 4. Discussion

This large-scale, population-based study confirms a significant association between AA and iron deficiency anemia (IDA). Our findings contribute to the growing understanding of AA as a systemic condition, extending beyond the confines of dermatological disease.

The relationship between AA and IDA has been debated for decades. Earlier studies, often limited by small sample sizes, heterogeneous populations, and cross-sectional designs, failed to demonstrate a consistent association [11,12]. In contrast, more recent large cohort studies and meta-analyses have supported this link [8,13,14], suggesting that methodological differences, including study power and adjustment for confounders, may partly explain prior discrepancies. Our findings align with this evolving body of evidence.

Several mechanisms may explain this relationship. Iron is essential for DNA synthesis, cell proliferation, and immune regulation [15]. Iron deficiency can impair immune competence, particularly T-cell function, potentially exacerbating the immune dysregulation that drives AA [15]. Chronic systemic inflammation, a feature common to many autoimmune diseases, can further disrupt iron metabolism, perpetuating a cycle of iron depletion and immune dysfunction [15].

Notably, our temporal analysis indicates that IDA precedes AA in a substantial proportion of cases. While this finding raises the possibility that iron deficiency may contribute to disease onset or exacerbation, it should be interpreted with caution, as observational data cannot establish causality. Supporting this, a recent large study demonstrated an increased incidence of autoimmune diseases following a diagnosis of IDA [16], particularly among women; however, this likely reflects shared underlying immune dysregulation rather than a direct causal relationship.

Interestingly, AA Israeli-born patients show a greater association with AA than controls in our study, which may correspond to a highly stressful lifestyle linked to occupation, army conscription, family dynamics and other social changes including immigration and relocation. On the other hand, this association could be confounded by different prevalences of chronic diseases and access to healthcare.

Available evidence regarding the effect of iron supplementation in alopecia areata is limited and heterogeneous, with some studies suggesting clinical improvement, particularly in patients with documented deficiency, while others have not demonstrated a consistent benefit. Further well-designed prospective studies are required to clarify its therapeutic role [17,18]. Iron deficiency is the most prevalent micronutrient deficiency globally, affecting approximately one in four individuals [19]. It contributes significantly to the global burden of disease, with consequences ranging from impaired cognitive and motor development in children to decreased resistance to infections as well as with androgenic alopecia, telogen effluvium and diffuse hair loss [20]. Therefore, assessment of iron status is a reasonable component of the AA work-up, particularly in populations at increased risk of iron deficiency, such as premenopausal women, adolescents and individuals experiencing heavy menstrual bleeding, dietary iron restriction, or coexisting autoimmune disease. Measurement of hemoglobin, serum ferritin, and transferring saturation may help identify a modifiable factor that may influence disease severity, persistence, or response to therapy. Correction of confirmed IDA is recommended for general health and may support hair follicle biology by improving anagen maintenance and follicular energy metabolism. Importantly, untreated iron deficiency may confound clinical assessment of alopecia areata activity or therapeutic response, particularly in patients receiving targeted immunomodulatory treatments.

The approval of JAK inhibitors for the treatment of AA [21,22,23] has significantly advanced the therapeutic landscape. However, these therapies carry potential risks and do not yield uniformly optimal outcomes. Given the role of iron in immune regulation and hair follicle biology, iron deficiency may represent a biologically relevant and potentially modifiable contributor to AA. In this context, its recognition in clinical practice may be particularly pertinent, as addressing such factors could complement overall disease management within a comprehensive and individualized care approach.

AA frequently coexists with other autoimmune diseases, including thyroiditis, vitiligo, rheumatoid arthritis, and celiac disease [7,8,9]. This clustering supports the concept of shared pathogenic pathways and underlines the importance of considering AA as part of a systemic autoimmune spectrum and underscore the multifaceted nature of the disease. These findings call for an integrated approach based on multidisciplinary care and comprehensive screening.

These findings may also have implications for the emerging paradigm of personalized medicine in dermatology. Identification of IDA in patients with AA may help stratify individuals who could benefit from targeted metabolic or nutritional interventions in addition to immunomodulatory therapy. Given the heterogeneity of AA in terms of clinical course and treatment response, incorporating iron status into routine evaluation may contribute to a more individualized management strategy. Screening for and correcting IDA represents a simple, low-risk intervention that may optimize patient outcomes and complement emerging targeted therapies such as JAK inhibitors.

Our study has several notable strengths, including a large sample size, a robust case–control design, and the use of systematically coded diagnoses. Nevertheless, certain limitations should be considered. The retrospective design precludes causal inference, and potential confounders—including chronic diseases, nutritional status and dietary factors, menstrual status, undiagnosed gastrointestinal blood loss, and coexisting autoimmune diseases—were not systematically available or controlled for in our dataset. Given the well-established association between alopecia areata and other autoimmune conditions, the absence of these data may represent an additional source of confounding in the observed relationship with iron deficiency anemia. Furthermore, reliance on coded diagnoses may introduce misclassification bias.

## 5. Conclusions

This study reinforces the significant association between AA and IDA and provides further support for considering AA as a systemic disease. Clinicians should be aware of this relationship and consider evaluating iron status and treating IDA in all AA patients, especially those with other autoimmune comorbidities or unexplained fatigue. Incorporating micronutrient profiling into AA management may therefore support a more comprehensive precision-medicine approach, allowing clinicians to tailor evaluation and treatment to individual patient risk profiles.

Finally, long term bi-directional studies are needed to determine whether early identification and correction of IDA can favorably influence the clinical course of AA and whether earlier and/or more aggressive treatment of AA may limit the development of comorbidities, in order to optimize the clinical outcome as well as identify the most cost-effective treatment.

## Figures and Tables

**Table 1:** Clinical and demographic characteristics of patients with alopecia areata and controls.

		Total (*n* = 100,203)		Control (*n* = 66,802)		Case (*n* = 33,401)		*p*-Value
		*n*	%	*N*	%	*n*	%	
Age (years) at diagnosis (mean ± standard deviation)		29.9 ± 16.9		29.9 ± 16.9		29.9 ± 16.9		1.000
Gender	Female	43,638	43.5	29,092	43.5	14,546	43.5	1.000
	Male	56,565	56.5	37,710	56.5	18,855	56.5	
Country of birth	Israel	82,873	82.7	53,829	80.6	29,044	87.0	<0.001
	Other	17,330	17.3	12,973	19.4	4357	13.0	

**Table 2:** Odds ratios for iron deficiency anemia in alopecia areata patients and controls in different age groups.

	Alopecia Areata	Iron Deficiency Anemia				OR (95%CI)	*p*-Value
		No		Yes			
		*n*	%	*n*	%		
All (*n* = 100,203)	Control 66,802	60,093	89.96	6709	10.04	1.61 [1.55–1.67]	<0.001
	Case 33,401	28,321	84.79	5080	15.21		
Age < 18 (*n* = 27,363)	Control 18,242	16,476	90.32	1766	9.68		
	Case 9121	7787	85.38	1334	14.62	1.60 (1.48–1.73)	<0.001
Age 18–40 (*n* = 44,169)	Control 29,446	26,931	91.46	2515	8.54		
	Case 14,723	12,756	86.64	1967	13.36	1.65 (1.55–1.76)	<0.001
Age > 40 (*n* = 28,671)	Control 19,114	16,686	87.3	2428	12.7		
	Case 9557	7778	81.39	1779	18.61	1.57 (1.47–1.58)	<0.001

## Data Availability

The original contributions presented in this study are included in the article. Further inquiries can be directed to the corresponding author.
